# Supplementary material for: Executioner caspases restrict mitochondrial RNA-driven Type I IFN induction during chemotherapy-induced apoptosis
Source: Nat Commun. 2023 Mar 14;14:1399. doi: 10.1038/s41467-023-37146-z (PMC10015073; doi:10.1038/s41467-023-37146-z)

Supplementary Information for

Executioner caspases restrict mitochondrial RNA-driven Type I IFN induction during chemotherapy-induced apoptosis

Shane T. Killarney[1], Rachel Washart[1], Ryan S. Soderquist[1], Jacob P. Hoj[1], Jamie Lebhar[1], Kevin H. Lin[1,2], Kris C. Wood[1]✉

[1]Department of Pharmacology and Cancer Biology, Duke University, Durham, NC, USA
[2]Present address: Department of Medicine, Brigham and Women's Hospital and Harvard Medical School, Boston, MA, USA
✉e-mail: kris.wood@duke.edu

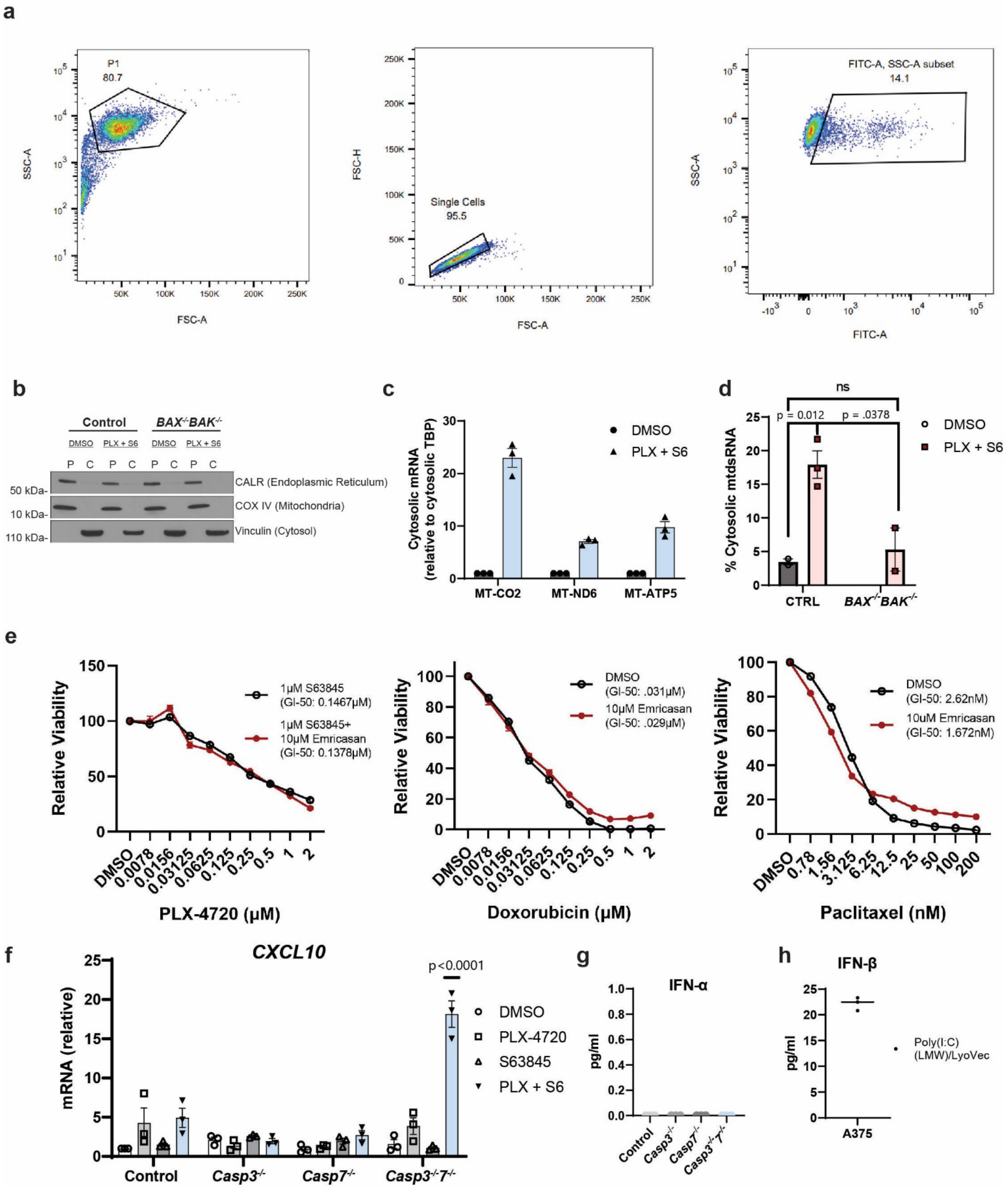

Supplementary Fig. 1: Caspase-independent cell death actives Type I IFN signaling. **a,** Flow cytometry gating strategy for data in Fig. 1b. **b,** Western blot from pellet (P) and cytosolic

(C) fractions following treatment of A375 wild-type cells with 0.5 μM PLX-4720, 0.5 μM S63845 for 36hrs; representative of three independent fractionation experiments (*n* = 3**). c,** A375 wild-type cells were treated with DMSO or PLX + S6 before RT-qPCR was performed on cytosolic fractions. A two-tailed unpaired t-test was performed, p-values are included in the figure. Data are presented as mean ± SEM. **d,** Quantification of cytosolic dsRNA species in A375 wild-type and *BAX*$^{-/-}$*BAK1*$^{-/-}$ upon DMSO or 0.5 μM PLX-4720, 0.5 μM S63845 treatment for 36hrs[62]. One-way ANOVA with Tukey's multiple-comparison test was performed, p-values are included in the figure. Data are presented as mean ± SEM. **e,** Relative cell viability of A375 cells following treatment with increasing doses of PLX-4720, doxorubicin, or paclitaxel in the presence or absence of 10 μM Emricasan. PLX-4720 treatment was performed with a background dose of 1 μM S63845. Data are presented as mean ± s.e.m for *n = 3* biologically independent experiments. **f,** A375 wild-type control, *CASP3*$^{-/-}$, *CASP7*$^{-/-}$, *CASP3*$^{-/-}$*7*$^{-/-}$ cells were treated with 0.5 μM PLX-4720, 0.5 μM S63845, or the combination (PLX + S6) for 24 hrs before performing RT-qPCR analysis of *CXCL10* expression. mRNA levels are normalized to DMSO-treated control cells; Two-way ANOVA with Tukey's multiple-comparisons test, p-values are included in the figure. Data are presented as mean ± SEM. **g,** ELISA analysis for IFN-α production in the media of A375 wild-type control, *CASP3*$^{-/-}$, *CASP7*$^{-/-}$, and *CASP3*$^{-/-}$*7*$^{-/-}$ cells following treatment with 2 μM PLX-4720 and 2 μM S63845 for 24 hrs. **h,** ELISA analysis for IFN-β in the supernatant of A375 cells treated with the 0.5 μg/ml of the cytosolic dsRNA agonist Poly (I:C) (LMW)/LyoVec for 12hrs. **c-d, and f-g** Each dot represents a biological replicate, and the data are the results of three independent experiments (*n* = 3).

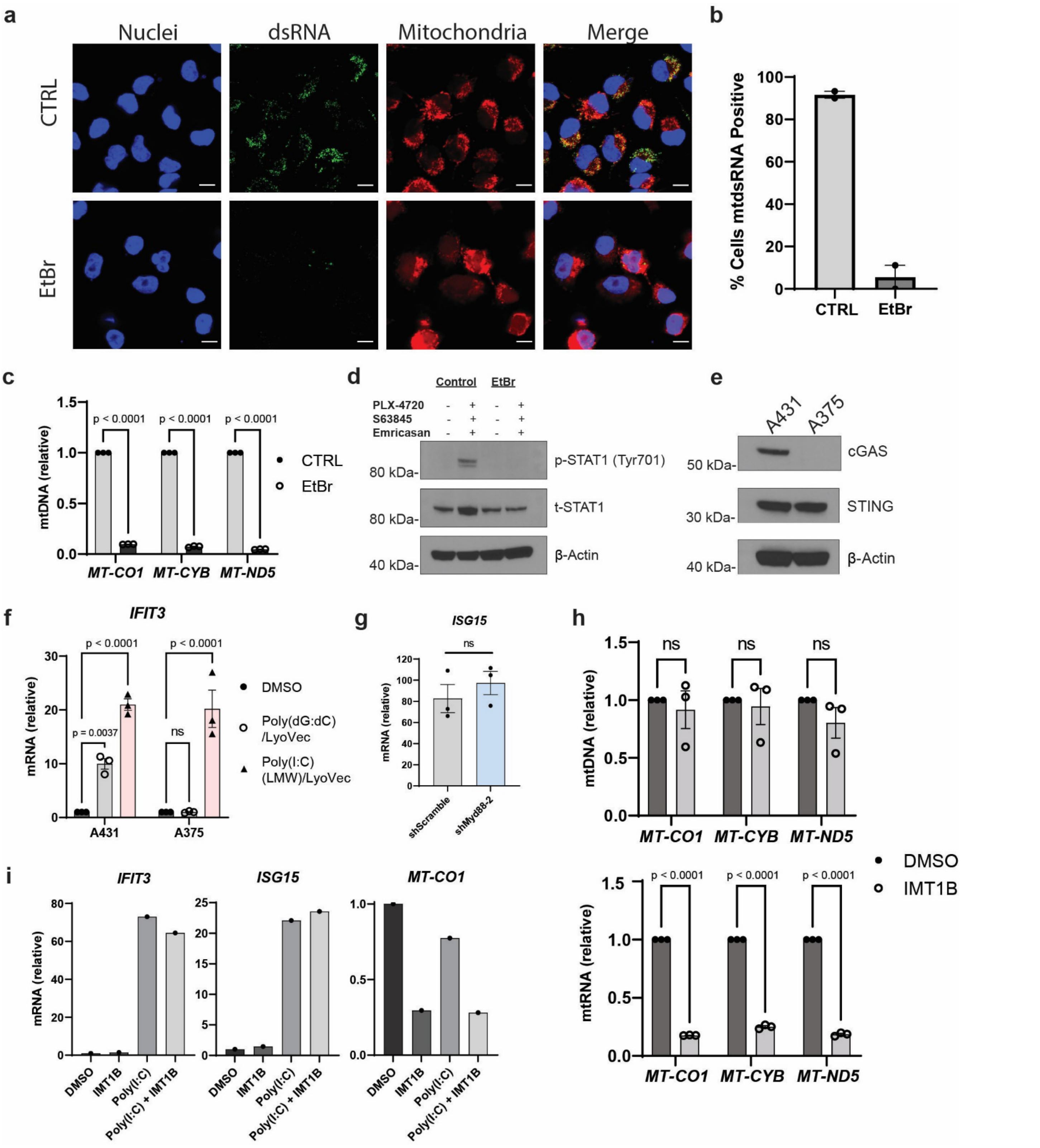

Supplemental Fig. 2: Validation of mtRNA depletion. a, A375 cells were pre-treated with EtBr (100 ng/ml) or control-media (CTRL) for 5-days before immunofluorescence images of

dsRNA with an anti-dsRNA (J2) antibody were taken. Mitochondria and nuclei are stained with MitoTracker Deep Red and Hoechst, respectively. Scale bars, 10 µm. Data are representative of two non-overlapping images. **b,** Graph representing the percentage of cells with mitochondrial dsRNA staining across two representative immunofluorescence images from experiment a. Data are presented as mean ± SEM. **c,** A375 cells were treated with EtBr (100 ng/ml) or control media for 5-days and qPCR was performed to assess mtDNA levels. **d,** Pre-treated EtBr, or control A375 cells were treated with the indicated combinations of PLX-4720 (0.5 µM), S63845 (0.5 µM), and Emricasan (10 µM) and a western blot of the specified proteins was performed. Data are representative of three independent experiments (n = 3) **e,** Western blot showing baseline ¬expression of indicated proteins in A431 and A375 cells. Data are representative of three independent experiments (n = 3). **f,** A431 and A375 cell lines were treated with 0.5 µg/mL of either Poly(dG:dC)/LyoVec or Poly(I:C) (LMW)/LyoVec for 16 hours. Following treatment, RT-qPCR analysis was performed to measure IFIT3 expression. mRNA was normalized to DMSO-treated control cells. One-way ANOVA with Tukey's multiple-comparisons test, p-values are included in the figure. Data are presented as mean ± SEM. **g,** A375 cells with tet-on hairpins against the indicated genes were pre-treated for 48hrs with 100 ng/ml of doxycycline before being subjected to either DMSO or PLX-4720, S63845, and Emricasan for 24hrs. RT-qPCR was normalized to DMSO treated cells. **h,** A375 cells were treated with 2.5 µM IMT1B for 24 hrs. The cells recovered from this experiment were split equally into two aliquots. The first aliquot was subject to qPCR measurement of mtDNA (top panel). RT-qPCR was performed on the second aliquot to measure the expression MT-CO1, MT-CYB, and MT-ND5 (bottom panel). mtDNA and mtRNA were normalized to their respective DMSO-treated control cells. **i,** A375 were treated with 10 µg/mL of the dsRNA agonist Poly(I:C) for 8 hrs in the presence or absence of 2.5 µM of IMT1B. RT-qPCR was performed on cell pellets from this experiment to measure mRNA expression of the indicated genes**.** Experiment was performed in singlicate (*n* = 1). **c, f, g, h,** two-tailed unpaired t-test, p-values are included in the figure. Data are presented as mean ± SEM. Each dot represents a biological replicate, and the data are the results of three independent experiments (*n* = 3).

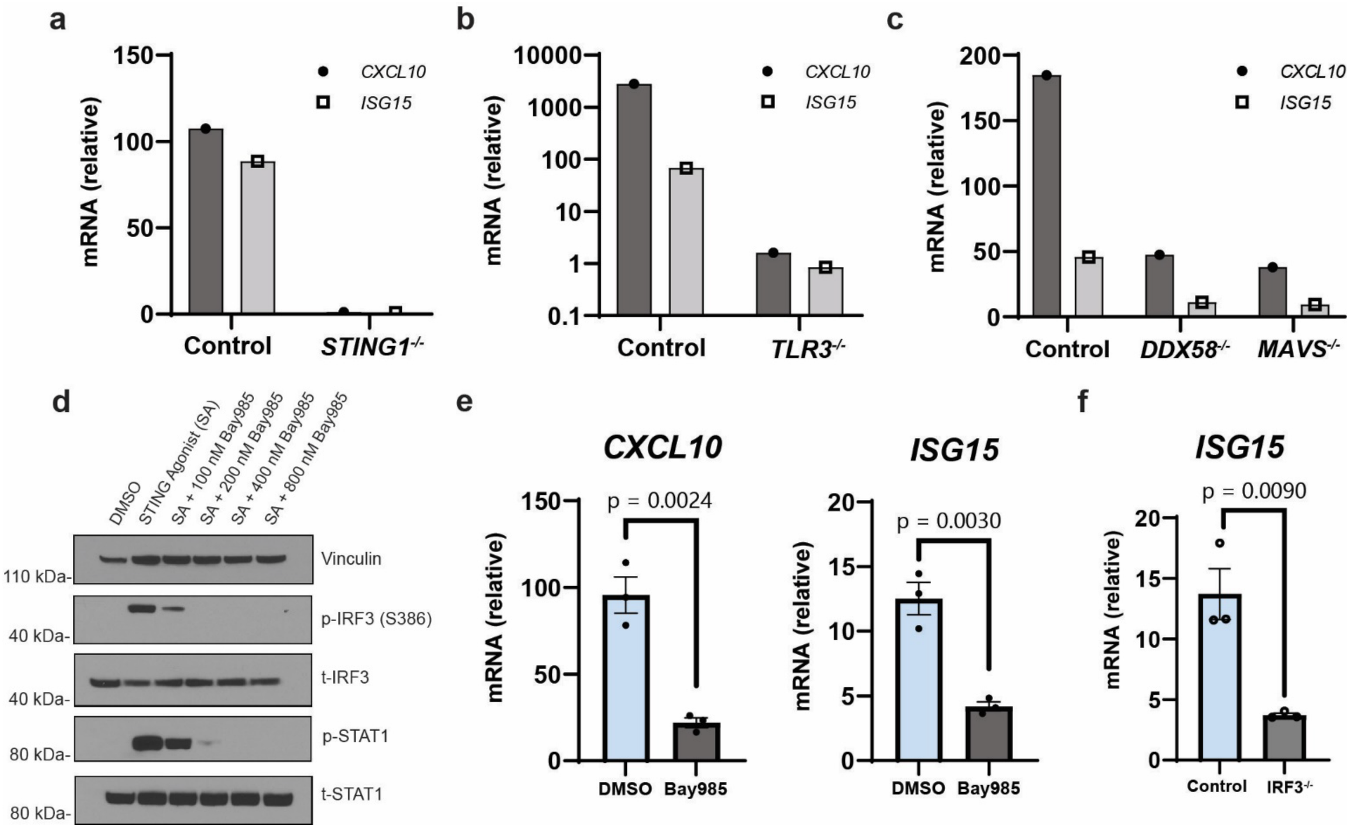

Supplementary Fig. 3: Cytosolic dsRNA sensing pathways control Type I IFN production during CICD. RT-qPCR was performed to measure the expression of *ISG15* and *CXCL10* expression in the following settings. **a,** A375 *CASP3*$^{-/-}$*7*$^{-/-}$ or *CASP3*$^{-/-}$*7*$^{-/-}$ + *STING*$^{-/-}$ cells treated with 0.5 µM STING agonist (diABZI compound 3) for 8 hrs. **b,** A375 *CASP3*$^{-/-}$*7*$^{-/-}$ or *CASP3*$^{-/-}$*7*$^{-/-}$ + *TLR3*$^{-/-}$ cells treated with 10 ug/ml poly(I:C) HMW for 16 hrs**. c,** A375 *CASP3*$^{-/-}$*7*$^{-/-}$, *CASP3*$^{-/-}$*7*$^{-/-}$ + *DDX58*$^{-/-}$, or *CASP3*$^{-/-}$*7*$^{-/-}$ + *MAVS*$^{-/-}$ cells treated with 0.5 µg/mL Poly(I:C) (LMW)/LyoVec for 16 hrs. **a, b, c** mRNA was normalized to DMSO-treated *CASP3*$^{-/-}$*7*$^{-/-}$ control cells. **d,** A375 wild-type cells were pre-treated with the indicated dose of Bay985 for one hour before an 8hr treatment with 0.5 µM diABZI (compound 3). Western blot showing the indicated proteins. Data are representative of three independent experiments (*n* = 3). **e,** A375 *CASP3*$^{-/-}$*7*$^{-/-}$ cells were treated with PLX-4720 (2 µM) and S63845 (2 µM) in the presence or absence of the TBK1/IKKε inhibitor Bay-985 (.2 µM) for 24 hrs. RT-qPCR analysis of *CXCL10* and *ISG15* was performed. **f,** A375 wild-type control or *IRF3*$^{-/-}$ cells following treatment with PLX-4720 (2 µM), S63845 (2 µM), and Emricasan (10 µM) for 24 hrs. RT-qPCR analysis *ISG15* was performed. **e,f,** Each dot represents a biological replicate, and the data are the results of three independent experiments (*n* = 3). Two-tailed unpaired t-test, p-values are included in the figure. Data are presented as mean ± SEM.

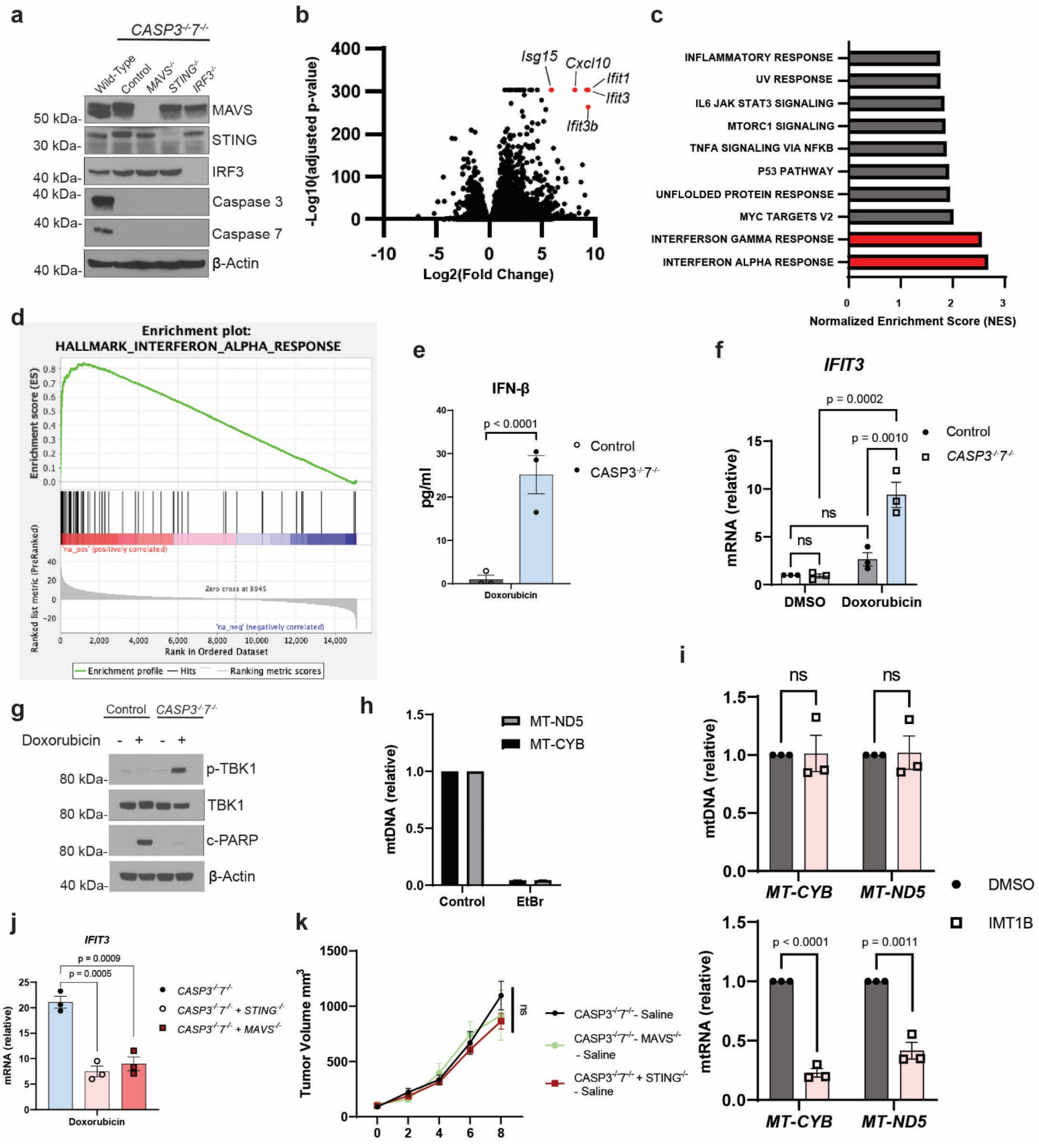

Supplemental Fig. 4: mtRNA stimulates therapeutic Type I IFN production during CICD in a murine melanoma model. a, Western blot from B16 cells with the indicated knockout to validate protein loss. Data are representative of three independent experiments (*n* = 3) **b,** RNA-seq analysis of differential expression in doxorubicin (0.5 μM) treated *CASP3*$^{-/-}$*7*$^{-/-}$ cells vs

doxorubicin treated (0.5 μM) wild-type control B16 cells at 72 hrs. Top hits are identified with a red dot and gene name. Statistical analysis is defined in the methods. **c,** Top ten gene sets enriched in the *CASP3*$^{-/-}$*7*$^{-/-}$ cells from experiment **b** are shown in order based on the normalized enrichment score (NES) from GSEA analysis. **d,** IFN-α GSEA plot from experiment **b** showing enriched genes in doxorubicin treated *CASP3*$^{-/-}$*7*$^{-/-}$ compared to doxorubicin treated wild-type control cells. **e,** ELISA analysis for IFN-β production in the media of B16 wild-type control and *CASP3*$^{-/-}$*7*$^{-/-}$ cells following treatment with 1.5 μM of doxorubicin for 24 hrs. Two-tailed unpaired t-test, p-values are included in the figure. Data are presented as mean ± SEM. **f,** B16 wild-type control and *CASP3*$^{-/-}$*7*$^{-/-}$ cells were treated with DMSO or 1.5 μM of doxorubicin for 24 hrs and RT-qPCR was performed to assess *IFIT3* expression. mRNA was normalized to DMSO-treated control cells. Two-way ANOVA with Tukey's multiple-comparisons test, p-values are included in the figure. Data are presented as mean ± SEM. **g,** Western blot showing the indicated proteins from B16 wild-type control and *CASP3*$^{-/-}$*7*$^{-/-}$ lysates that were treated with 1.5 μM doxorubicin for 24 hrs and the data are the results of three independent experiments (*n* = 3). **h,** B16 CASP3$^{-/-}$7$^{-/-}$ cells were treated with EtBr (100 ng/ml) or control media for 5-days and qPCR was performed to assess mtDNA levels. **i,** B16 cells were treated with 2.5 μM IMT1B for 24 hrs. The cells recovered from this experiment were split equally into two aliquots. The first aliquot was subject to qPCR measurement of mtDNA (top panel). RT-qPCR was performed on the second aliquot to measure the expression *MT-CYB* and *MT-ND5* (bottom panel). mtDNA and mtRNA were normalized to their respective DMSO-treated control cells. Two-tailed unpaired t test, p-values are included in the figure. Data are presented as mean ± SEM. **j,** RT-qPCR analysis of *IFIT3* expression in B16 CASP3$^{-/-}$7$^{-/-}$ cells with additional knockouts against STING or MAVS following treatment of 1.5 μM doxorubicin for 24 hrs. Each dot represents a biological replicate, and the data are the results of three independent experiments (*n* = 3). One-way ANOVA with Tukey's multiple-comparisons test, p-values included in the figure. Data are presented as mean ± SEM. **k,** Tumor volume growth curves after subcutaneous injection of B16 *CASP3*$^{-/-}$*7*$^{-/-}$ cells with an additional knockout against STING or MAVS in the flanks of C57BL/6 mice treated with either doxorubicin (Dox) or saline. Two-way ANOVA with Tukey's multiple-comparison test. Data are presented as mean ± SEM.

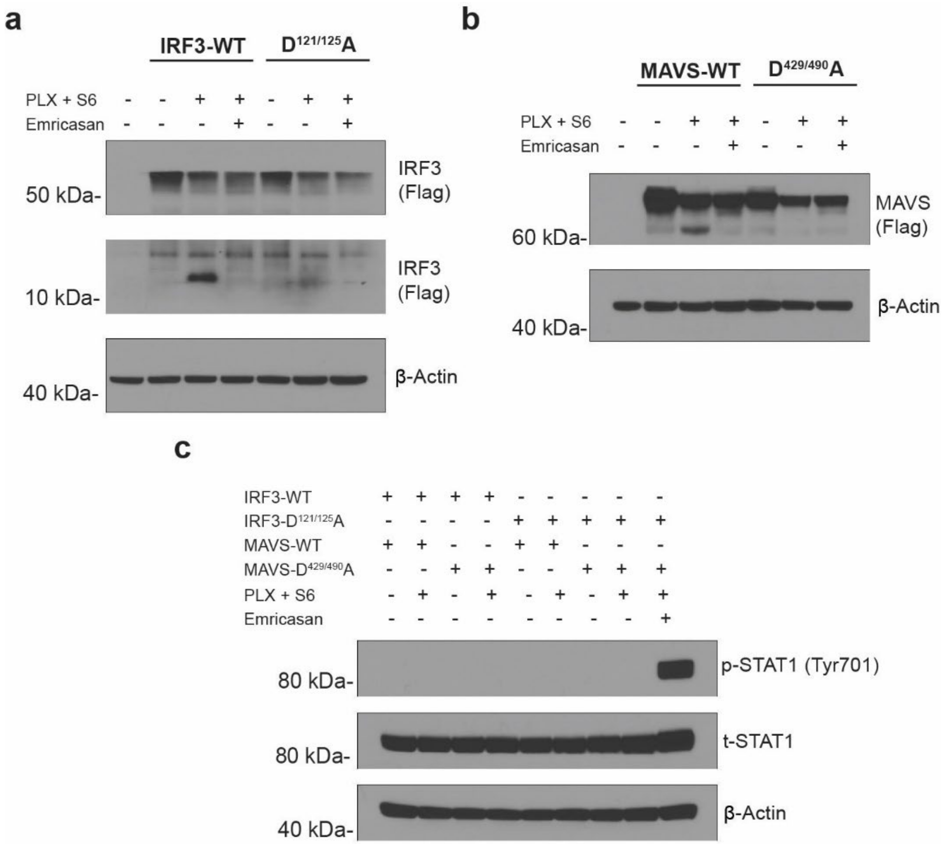

Supplemental Fig. 5: Apoptotic caspases cleave IRF3 and MAVS. a, A375 wild-type cells expressing flag-tagged IRF3 wild-type (IRF3-WT), flag-tagged IRF3 $D^{121/125}A$ mutant, or empty backbone (lane 1) were treated with the indicated combinations of 2 μM PLX-4720 (PLX), 2 μM S63845 (S6), and 10 μM Emricasan for 24 hrs before immunoblotting was performed. **b,** A375 wild-type cells expressing flag-tagged MAVS wild-type (MAVS-WT), flag-tagged MAVS $D^{429/490}A$ mutant, or empty backbone (lane 1) were treated with the indicated combinations of 2 μM PLX-4720 (PLX), 2 μM S63845 (S6), and 10 μM Emricasan for 24 hrs before immunoblotting was performed. **c,** A375 wild-type cells with stable expression of the indicated combinations of IRF3-WT, IRF3-$D^{121/125}A$, MAVS-WT, and MAVS-$D^{429/490}A$ were treated with DMSO or 2 μM PLX-4720 (PLX) and 2 μM S63845 (S6) for 24 hrs before immunoblotting. PLX + S6 + 10 μM Emricasan was used as a positive control for p-STAT1 activity. **a-c,** Data are representative of three independent experiments (*n* = 3).

Supplemental Fig. 1b: Raw Blot

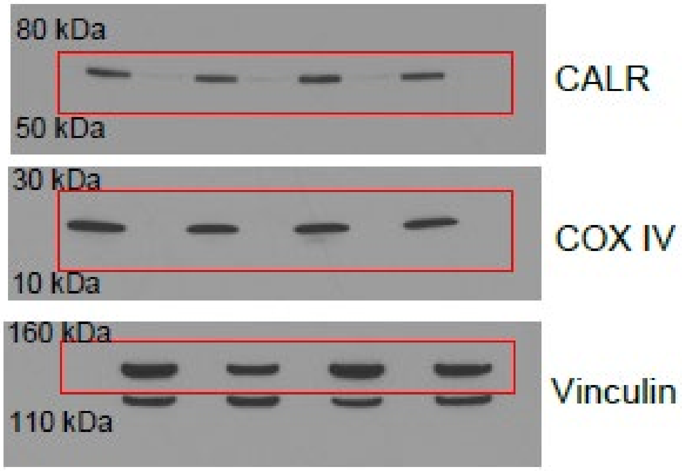

Supplemental Fig. 2d: Raw Blots

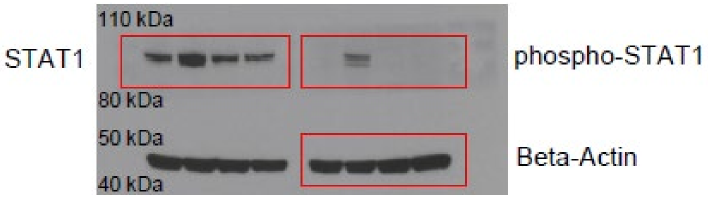

Supplemental Fig. 2e: Raw Blots

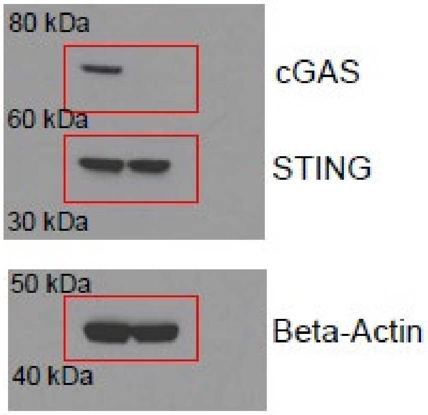

Supplemental Fig. 3d: Raw Blot

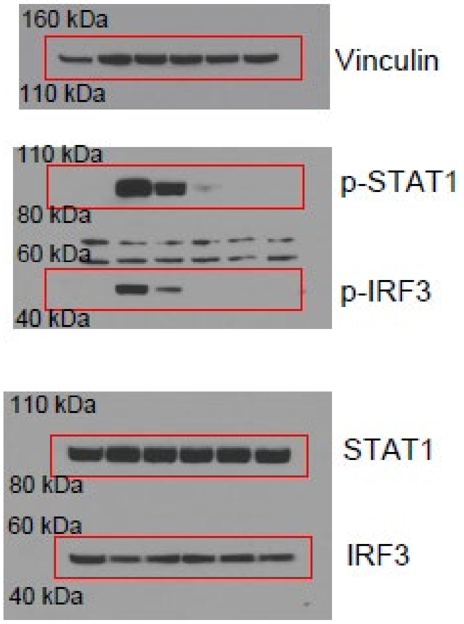

Supplemental Fig. 4a: Raw Blot

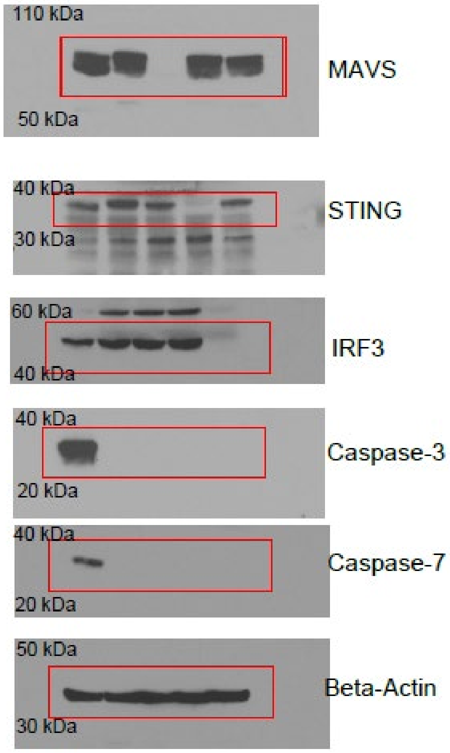

Supplemental Fig. 4g: Raw Blot

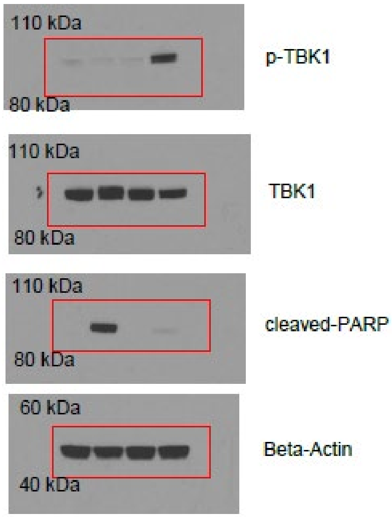

Supplemental Fig. 5a: Raw Blot

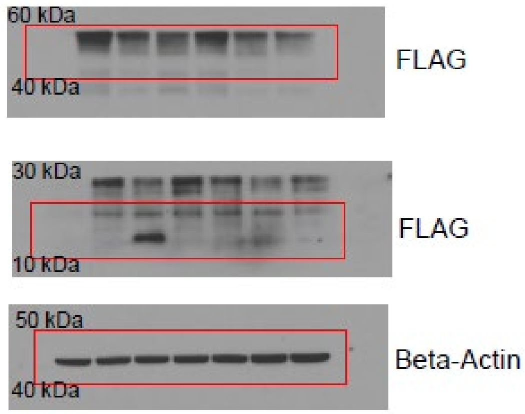

Supplemental Fig. 5b: Raw Blot

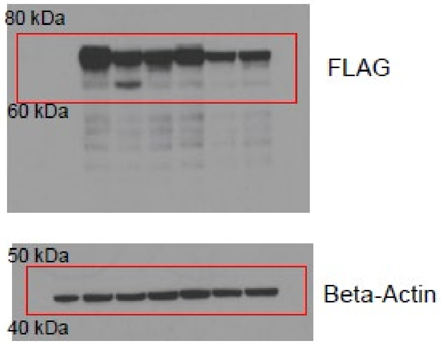

Supplemental Fig. 5c: Raw Blot

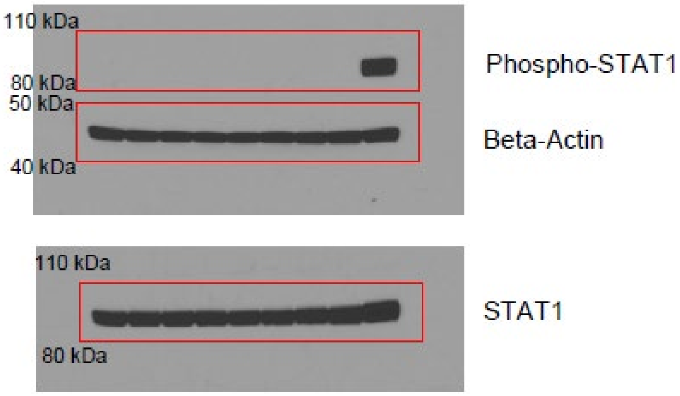

Supplement: Supplementary file 1 — Supplementary Information [file 41467_2023_37146_MOESM1_ESM.pdf]
