# Supplementary material for: Executioner caspases restrict mitochondrial RNA-driven Type I IFN induction during chemotherapy-induced apoptosis
Source: Nat Commun. 2023 Mar 14;14:1399. doi: 10.1038/s41467-023-37146-z (PMC10015073; doi:10.1038/s41467-023-37146-z)
Supplement: Supplementary file 2 — Reporting Summary [file 41467_2023_37146_MOESM2_ESM.pdf]

nature portfolio

Corresponding author(s): Kris C. Wood

Last updated by author(s): Feb 7, 2023

Reporting Summary

Nature Portfolio wishes to improve the reproducibility of the work that we publish. This form provides structure for consistency and transparency in reporting. For further information on Nature Portfolio policies, see our Editorial Policies and the Editorial Policy Checklist.

Statistics

For all statistical analyses, confirm that the following items are present in the figure legend, table legend, main text, or Methods section.

n/a	Confirmed	
☐	☒	The exact sample size (*n*) for each experimental group/condition, given as a discrete number and unit of measurement
☐	☒	A statement on whether measurements were taken from distinct samples or whether the same sample was measured repeatedly
☐	☒	The statistical test(s) used AND whether they are one- or two-sided<br>*Only common tests should be described solely by name; describe more complex techniques in the Methods section.*
☒	☐	A description of all covariates tested
☐	☒	A description of any assumptions or corrections, such as tests of normality and adjustment for multiple comparisons
☐	☒	A full description of the statistical parameters including central tendency (e.g. means) or other basic estimates (e.g. regression coefficient) AND variation (e.g. standard deviation) or associated estimates of uncertainty (e.g. confidence intervals)
☐	☒	For null hypothesis testing, the test statistic (e.g. *F*, *t*, *r*) with confidence intervals, effect sizes, degrees of freedom and *P* value noted<br>*Give P values as exact values whenever suitable.*
☒	☐	For Bayesian analysis, information on the choice of priors and Markov chain Monte Carlo settings
☒	☐	For hierarchical and complex designs, identification of the appropriate level for tests and full reporting of outcomes
☒	☐	Estimates of effect sizes (e.g. Cohen's *d*, Pearson's *r*), indicating how they were calculated

Our web collection on statistics for biologists contains articles on many of the points above.

Software and code

Policy information about availability of computer code

Data collection	-Real-time qPCR: CFX384 Touch Real-Time PCR Detection System<br>-Cell Counting: Z2 Coulter Particle Count and Size Analyzer<br>-Plate Reader for luminescence assays: TECAN infinite M1000Pro<br>-Blot image scanning: Epson Perfection V600 Photo<br>-DNA/RNA concentration measurement: Thermo Fisher NanoDrop Lite<br>-FACS Analysis: Becton Dickinson FACSCanto II and Beckman Coulter Astrious Cell Sorter
Data analysis	-Statistical analysis and plotting were done in GraphPad Prism 8.0.2, 9.0.1 or Microsoft Excel 2016-2021<br>-Figures were prepared in Adobe Illustrator CC 2017, 2020<br>-Western blot images were scanned with EPSON Scan 3.9.4<br>-Flow cytometry data were analyzed in Imagej 1.51<br>-RNAseq data was analyzed with GSEA software (v4.1.0, The Broad Institute), RStudio Version 1.1.463, 1.3.1073, and R version 3.5.1, 4.0.2. The following R packages were used "DESeq2" and "JACoP"

For manuscripts utilizing custom algorithms or software that are central to the research but not yet described in published literature, software must be made available to editors and reviewers. We strongly encourage code deposition in a community repository (e.g. GitHub). See the Nature Portfolio guidelines for submitting code & software for further information.

Data

Policy information about availability of data

All manuscripts must include a data availability statement. This statement should provide the following information, where applicable:

- Accession codes, unique identifiers, or web links for publicly available datasets
- A description of any restrictions on data availability
- For clinical datasets or third party data, please ensure that the statement adheres to our policy

All data associated with this study are available in the main text or the supplementary materials. Source data are provided with this paper. RNA-seq data from B16 sgCTRL and sgCasp3/7 cells treated with doxorubicin and reads were mapped to the mouse genome (GRCm38) using the HISAT2 alignment tools. The data is available at Gene Expression Omnibus (GEO) (Accession Number GSE210377).

Human research participants

Policy information about studies involving human research participants and Sex and Gender in Research.

Reporting on sex and gender	N/A
Population characteristics	N/A
Recruitment	N/A
Ethics oversight	N/A

Note that full information on the approval of the study protocol must also be provided in the manuscript.

Field-specific reporting

Please select the one below that is the best fit for your research. If you are not sure, read the appropriate sections before making your selection.

[x] Life sciences [ ] Behavioural & social sciences [ ] Ecological, evolutionary & environmental sciences

For a reference copy of the document with all sections, see nature.com/documents/nr-reporting-summary-flat.pdf

Life sciences study design

All studies must disclose on these points even when the disclosure is negative.

Sample size	As described, in vitro sample sizes ranged from n=3 to n=5 replicates per condition. Sample sizes were sufficient to identify significant changes as indicated in each figure.<br><br>For mouse studies no sample size estimation experiment was initially performed. The sample size were based upon expected variability and effect sizes for similar tumor models with other treatments and confirmed to be in line with our models effect size:<br>Han, C. et al. Tumor cells suppress radiation-induced immunity by hijacking caspase 9 signaling. Nat. Immunol. 21, 546–554 (2020).
Data exclusions	No data were excluded from analysis.
Replication	Experimental findings were performed at least 3 independent times. The experimental findings were reliably reproduced and all attempts were included in the presentation unless technical error prevented the completion of the experiment.
Randomization	Mice were randomized upon their tumor volume reaching 50 mm^3. No randomization was required for the in vitro cell line experimentation.
Blinding	Blinding the in vivo experiments was not feasible given the labeling requirements of the facilities and limitation of available personnel. To establish humane endpoint for mouse study, blinded observers visually inspected mice for obvious signs of distress, such as loss of appetite, hunched posture.No blinding of in vitro experiments was performed as each cell line requires different culture conditions.

Reporting for specific materials, systems and methods

We require information from authors about some types of materials, experimental systems and methods used in many studies. Here, indicate whether each material, system or method listed is relevant to your study. If you are not sure if a list item applies to your research, read the appropriate section before selecting a response.

Materials & experimental systems

n/a	Involved in the study
☐	☒ Antibodies
☐	☒ Eukaryotic cell lines
☒	☐ Palaeontology and archaeology
☐	☒ Animals and other organisms
☒	☐ Clinical data
☒	☐ Dual use research of concern

Methods

n/a	Involved in the study
☒	☐ ChIP-seq
☐	☒ Flow cytometry
☒	☐ MRI-based neuroimaging

Antibodies

Antibodies used

Cell Signaling: PARP (#9532), Cleaved Caspase-3 (Asp175) (#9661), Cleaved Caspase-7 (Asp198), BAX (#2772), BAK (#12105), β-Actin (#4970), Calreticulin (#12238), COX IV (#4844), Vinculin (#13901S), phospho-Stat1 (Tyr701) (#9167), Stat1 (#9172), Caspase-9 (#9502), Caspase-3 (#9662), Caspase-7 (#12827), STING (#13647), MDA-5 (#5321), RIG-I (#3743), MAVS (#3993), MAVS (#83000), Toll-like Receptor 3 (#6961), Phospho-IRF-3 (Ser386) (#37829), IRF-3 (#4302), Phospho-TBK1/NAK (Ser172) (#5483), TBK1/NAK (#3504), cGAS (#79978), and Cleaved PARP (#9548). Monoclonal ANTI-FLAG® M2 antibody (F3165) was purchased from Sigma. Mouse anti double-stranded RNA J2 (exalpha 10010500). All antibodies were used at 1:1000 dilution for western blotting. Anti-J2 was used at 2.5 μg/ml for IF.

Validation

Antibody validation of the manufacturers were available for all antibodies including positive and negative staining controls. Additionally we confirmed BAX (#2772), BAK (#12105), Caspase-3 (#9662), Caspase-7 (#12827), STING (#13647), MDA-5 (#5321), RIG-I (#3743), MAVS (#3993), MAVS (#83000), Toll-like Receptor 3 (#6961), Phospho-IRF-3 (Ser386) (#37829), IRF-3 (#4302), and cGAS (#79978) through knockout experiments. Anti-J2 (exalpha 10010500) was validated with mtDNA depletion. PARP (#9532), Cleaved Caspase-3 (Asp175) (#9661), Cleaved Caspase-7 (Asp198), phospho-Stat1 (Tyr701), and Phospho-TBK1/NAK (Ser172) (#5483) antibodies were experimentally validated with positive control experiments.

Eukaryotic cell lines

Policy information about cell lines and Sex and Gender in Research

Cell line source(s)

All cell lines were purchased from American Type Culture Collection (ATCC) or Duke University Cell Culture Facility (CCF). The commonly-used cancer cell lines used in this study were A375, Colo205, C32, A431, SK-MEL-28, and B16F10.

Authentication

All cell lines were authenticated prior to use using STR profiling.

Mycoplasma contamination

All cell lines were confirmed as mycoplasma-free upon receipt.

Commonly misidentified lines (See ICLAC register)

No cell lines were misclassified.

Animals and other research organisms

Policy information about studies involving animals; ARRIVE guidelines recommended for reporting animal research, and Sex and Gender in Research

Laboratory animals

Syngenic B16 tumors: Mus Musculus, 5-6 week old, C57BL/6 and were housed in the Duke Cancer Center Isolation Facility (CCIF). CCIF Environment: The facility is organized into 8 modules, each having multiple anterooms/airlocks and pass-through sterilizers. Modules 1-4 functions as single corridor modules with pass-through autoclaves and anterooms/airlocks, and modules 5-8 are organized on a clean-dirty pass-through system via anteroom/airlocks and corridors. Temperature, humidity and pressures are controlled by a pneumatic control system with digital backup alarms. Each module is provided with 100% outside air. Exhaust systems and Air supply is HEPA filtered. Species-specific heat and humidity are maintained within the parameters outlined in The Guide for the Care and Use of Laboratory Animals. The animals are fed a standard laboratory diet. Breeding or specialty diets will be provided upon request. Mice are housed in micro-isolated caging on enrich-o-corn cob bedding and changed every two weeks. Rats are changed once a week. DLAR will provide alternative bedding based on scientific need. Group housing of up to 5 mice per cage and up to 2 rats per cage is strongly encouraged. Husbandry practices are in accordance with "The Guide." Environmental enrichment for singly housed animals will be provided unless an exception is granted by the IACUC. All rodents in CCIF is housed in individually ventilated caging. DLAR provides disposable carriers and bags for transporting animals. In the event of overcrowding, investigators will be given 48 hour notice to separate the animals into new caging. Euthanasia chambers are available in each of the modules and carcass freezers are located in 105COR hallway. No BSL or hazardous agents should be administered to animals in CR II without prior consent from DLAR.

Wild animals

No wild animals were used in this study.

Reporting on sex

B16 mouse melanoma cells were extracted from a female mouse. We used female C57BL/6 mice for all of our studies to minimize any immune effects related to sex differences.

Field-collected samples

No samples collected from the field were used in this study.

Ethics oversight	The Duke University Institutional Animal Care & Use Committee (IACUC) reviewed and approved the cell line xenograft transplantation and treatment protocol described in this study.

Note that full information on the approval of the study protocol must also be provided in the manuscript.

Flow Cytometry

Plots

Confirm that:

- [x] The axis labels state the marker and fluorochrome used (e.g. CD4-FITC).
- [x] The axis scales are clearly visible. Include numbers along axes only for bottom left plot of group (a 'group' is an analysis of identical markers).
- [x] All plots are contour plots with outliers or pseudocolor plots.
- [x] A numerical value for number of cells or percentage (with statistics) is provided.

Methodology

Sample preparation	Samples were prepared according to the CellEvent™ Caspase-3/7 Green Detection Reagent (#C10423) manufacturer protocol.
Instrument	Becton Dickinson FACSCanto II
Software	FlowJo 2019
Cell population abundance	Cell populations were entirely pure of any contaminants.
Gating strategy	ells were gated for live cells based on FSC/SSC and singlets based on FSC.

- [x] Tick this box to confirm that a figure exemplifying the gating strategy is provided in the Supplementary Information.
